# Low-dose MDCT: evaluation of the impact of systematic tube current reduction and sparse sampling on the detection of degenerative spine diseases

**DOI:** 10.1007/s00330-020-07278-7

**Published:** 2020-09-18

**Authors:** Nico Sollmann, Kai Mei, Isabelle Riederer, Monika Probst, Maximilian T. Löffler, Jan S. Kirschke, Peter B. Noël, Thomas Baum

**Affiliations:** 1 Department of Diagnostic and Interventional Neuroradiology, Klinikum rechts der Isar, Technische Universität München, Ismaninger Str. 22, 81675 Munich, Germany; 2 TUM-Neuroimaging Center, Klinikum rechts der Isar, Technische Universität München, Munich, Germany; 3 Department of Diagnostic and Interventional Radiology, Klinikum rechts der Isar, Technische Universität München, Ismaninger Str. 22, 81675 Munich, Germany; 4 Department of Radiology, Perelman School of Medicine, University of Pennsylvania, 3400 Spruce Street, One Silverstein, Philadelphia, PA 19104, USA

**Keywords:** Artifacts, Intervertebral disc disease, Intervertebral disc degeneration, Image processing, computer-assisted, Radiation dosage

## Abstract

**Objectives:**

To investigate potential radiation dose reduction for multi-detector computed tomography (MDCT) exams of the spine by using sparse sampling and virtually lowered tube currents combined with statistical iterative reconstruction (SIR).

**Methods:**

MDCT data of 26 patients (68.9 ± 11.7 years, 42.3% males) were retrospectively simulated as if the scans were acquired at 50%, 10%, 5%, and 3% of the original X-ray tube current or number of projections, using SIR for image reconstructions. Two readers performed qualitative image evaluation considering overall image quality, artifacts, and contrast and determined the number and type of degenerative changes. Scoring was compared between readers and virtual low-dose and sparse-sampled MDCT, respectively.

**Results:**

Image quality and contrast decreased with virtual lowering of tube current and sparse sampling, but all degenerative changes were correctly detected in MDCT with 50% of tube current as well as MDCT with 50% of projections. Sparse-sampled MDCT with only 10% of initial projections still enabled correct identification of all degenerative changes, in contrast to MDCT with virtual tube current reduction by 90% where non-calcified disc herniations were frequently missed (R1: 23.1%, R2: 21.2% non-diagnosed herniations). The average volumetric CT dose index (CTDI_vol_) was 1.4 mGy for MDCT with 10% of initial projections, compared with 13.8 mGy for standard-dose imaging.

**Conclusions:**

MDCT with 50% of original tube current or projections using SIR still allowed for accurate diagnosis of degenerative changes. Sparse sampling may be more promising for further radiation dose reductions since no degenerative changes were missed with 10% of initial projections.

**Key Points:**

*• Most common degenerative changes of the spine can be diagnosed in multi-detector CT with 50% of tube current or number of projections.*

• *Sparse-sampled multi-detector CT with only 10% of initial projections still enables correct identification of degenerative changes, in contrast to imaging with 10% of original tube current.*

• *Sparse sampling may be a promising option for distinct lowering of radiation dose, reducing the CTDI*_*vol*_ *from 13.8 to 1.4 mGy in the study cohort.*

## Introduction

Back pain with or without radiculopathy has high prevalence worldwide and is a major reason for seeking medical advice [1–3]. Magnetic resonance imaging (MRI) is considered the imaging modality of choice for patients with refractory back pain, but computed tomography (CT) still is at the forefront of imaging particularly when rapid MRI is not available or contraindicated. CT can also act complementary to MRI, thanks to its potential to visualize mineralized bone with high contrast, providing additional valuable information to MRI in case of inconclusive findings. CT has proven to be accurate in cases of spinal or neural foraminal stenosis, particularly for distinguishing soft disc herniation from osteophytes, and it can depict bony lysis zones [4–6]. However, diagnostic CT of the spine exposes the patient to a considerable radiation dose, with radiation due to CT imaging showing drastic rises over the recent years [7, 8]. Conventional imaging by CT can entail estimated effective doses of approximately 5.6 mSv and 10.0 mSv for the lumbar and whole dorsal spine, respectively [9, 10]. Such radiation exposure as applied during CT acquisition is correlated with increased estimated cancer risk ratios that need to be considered in the light of patient safety [9–11].

Over the recent decades, several approaches on both the image acquisition side and image reconstruction side have been further developed for CT to lower radiation exposure while keeping image quality sufficient for diagnostic purposes. At the cervical spine, low-dose (LD) multi-detector CT (MDCT) achieved by lowering of tube currents was compared with standard-dose (SD) imaging using filtered back projection (FBP) and hybrid iterative reconstruction (IR), showing that LD imaging with IR provides better image quality for intervertebral discs, neural foramina, and ligaments, and worse image quality for soft tissues and vertebrae when compared with SD imaging with FBP in patients with chronic cervical pain and/or radiculopathy [12]. Another study used LD MDCT with FBP and hybrid IR, showing that high strength levels of IR are favorable for the intervertebral discs and the content of neural foramina at the cervical spine [13]. At the lumbar spine in patients with back pain, LD CT using model-based IR showed better tissue differentiation than hybrid IR or SD CT with FBP [14]. For patients with low to moderate body mass index and chronic back pain, ultra-LD CT protocols with hybrid IR have been tested, indicating preserved image quality and diagnostic accuracy [15]. Furthermore, an earlier study performed systematic simulations of lowered tube currents, indicating that tube charge settings could be reduced to 65% of SD imaging for diagnostics in patients with suspected lumbar disc herniation [16].

The small body of referenced previous studies on LD CT in patients with back pain with or without radiculopathy achieved reduced radiation exposure by lowering tube currents, partially combined with IR algorithms [12–16]. Modern advances like sparse sampling, a novel technique that is referred to as the acquisition of fewer projection images during scanning, have not been evaluated for diagnostic CT of the degenerative spine. However, sparse sampling reflects a promising alternative since the energy delivery for the individual projection image is maintained while overall radiation exposure is lowered as a consequence of decreased projection numbers [17, 18]. This can result in preserved image quality while the influence of electronic readout noise on image quality can be circumvented. When combined with advanced image reconstruction, such as statistical IR (SIR), image noise could be further suppressed and radiation doses lowered while structural image information can be preserved [17, 19–23].

Against this background, the present study investigates the potential of sparse-sampled MDCT combined with SIR to reduce radiation exposure for imaging of the degenerative spine. We hypothesized that sparse-sampled MDCT enables greater reductions in radiation dose when compared with MDCT with simulated lowered tube currents while delivering images with sufficient diagnostic quality.

## Materials and methods

### Study design and patient inclusion

This retrospective study was approved by the local institutional review board and was conducted in accordance with the Declaration of Helsinki. Patients who underwent non-contrast MDCT imaging of the spine at our department according to clinical indication (suspected degenerative spine disease or follow-up in degenerative spine disease) during a period of 1 month (June/July 2019) were identified in our hospital’s picture archiving and communication system (PACS). Exclusion criteria were (1) age below 18 years, (2) motion artifacts in imaging data, (3) previous surgery with instrumentation at the spine, (4) presence of any implants in the field of view (FOV), and (5) vertebral fractures, malignant bone lesions, or spondylodiscitic lesions captured by the FOV. Overall, 26 patients were eligible and included in this study.

### Imaging by multi-detector computed tomography

Image acquisition was performed in supine position using a 128-slice MDCT scanner (Ingenuity Core 128, Philips Healthcare). An initial scout scan was used for planning of the FOV, and subsequent helical scanning was acquired with implicit tube current modulation. Table 1 shows scanning details for MDCT imaging.

**Table 1:** Scanning details and image reconstruction

Scanning details and image reconstruction	
Tube voltage (in kV)	123.1 ± 7.2 (120.0–140.0)
Tube current (in mA)	321.5 ± 7.5 (309.0–334.0)
Rotation time (in s)	0.78 ± 0.163 (0.5, 0.75, or 1)
Exposure (in mAs)	194.5 ± 56.8 (130.0–314.0)
Voxel spacing (in mm^3^)	0.39 × 0.39 × 0.90
DLP (in mGy*cm)	388.9 ± 179.9 (45.5–782.1)
Field of view (in mm^2^)	200 × 200
Slice thickness (in mm)	0.9
Reformations	Sagittal, axial, coronal
Windowing	Individually adjustable—standard setting: window width 2500 HU, window center 500 HU (default setting for bone window)

### Simulations and image reconstruction

#### Tube current reduction

Initial preprocessing of imaging data used a total-variation method for the projection data to reduce image noise (*λ* = 0.01, *n* = 50) [24, 25]. By the use of a simulation algorithm based on raw imaging data, we generated MDCT scans with virtually lowered tube currents in a stepwise fashion [26–31]. The approach for simulations of LD MDCT has been validated previously [31]. Hence, simulations were generated as if MDCT was conducted with 50% (D50P100), 10% (D10P100), 5% (D5P100), and 3% (D3P100) of the original X-ray tube current. The original imaging data was defined as D100P100.

#### Sparse sampling

Sparse sampling was simulated by reading only a reduced amount of projection angles and by deleting the remaining projections in the sinogram [27–29, 32]. The original imaging was defined as D100P100, and virtual sparse-sampled imaging was generated as if MDCT was performed with only 50% (D100P50), 10% (D100P10), 5% (D100P5), and 3% (D100P3) of the original projection data.

#### Statistical iterative reconstruction

For image reconstruction of simulated MDCT with lowered tube current or sparse sampling, we used the same in-house developed SIR algorithm that was based on ordered-subset separable paraboloidal surrogate combining a momentum accelerating approach [33, 34]. A Gaussian noise model was applied and the likelihood term for SIR was computed with log-converted projection data. To enhance convergence and to further depress image noise while achieving adequate bone/soft tissue contrast, a regularization term based on a Huber penalty was applied. The distinct strength of the regularization term was selected in consensus with three board-certified radiologists. Linear attenuation coefficients of resulting imaging data were translated to Hounsfield units by using air and water information from the MDCT calibration data.

### Qualitative image analysis

Qualitative image evaluation was performed using the PACS viewer (IDS7, Sectra AB). Two radiologists (reader 1 [R1] and reader 2 [R2], 7 years of experience in radiology each) systematically assessed all reconstructed imaging data in all patients (D100P100, D50P100, D10P100, D5P100, D3P100, D100P50, D100P10, D100P5, and D100P3). Evaluations were performed after patient pseudonymization, and the readers had no access to the clinical reports for original imaging and were unaware of the distinct clinical indication that resulted in MDCT imaging. The readers evaluated the SD scan (D100P100) in consensus reading. All other imaging data were assessed separately, with the readers being strictly blinded to the ratings of each other. In detail, the readers performed evaluations of LD scans in the context of eight reading rounds, with an interval of at least 1 week between each round. Within each round, one reconstructed dataset of each patient was evaluated, with the distinct dataset shown per round being subject to randomization. Furthermore, the order of patient cases was also randomized per reading round.

Overall image quality, overall artifacts, and image contrast were evaluated first based on 5-point Likert scales considering the entire FOV (Table 2). Additionally, the readers performed segment-wise evaluation of degenerative changes to determine the presence or absence of such changes (dichotomous evaluation). Furthermore, in case of detected degenerative changes, the readers had to specify them per segment considering spondylosis, pseudospondylolisthesis, spondylolisthesis (with spondylolysis), non-calcified disc herniation, and disc herniation with calcification. In case of presence of more than one of the mentioned degenerative changes, the readers were requested to provide all segment-specific degenerative changes.

**Table 2:** Scoring scheme for qualitative image analysis

Qualitative image analysis					
Item	Score				
	1	2	3	4	5
Overall image quality	Very good to perfect quality	Good to very good quality	Medium quality	Poor quality	Inappropriate quality
Overall artifacts	No artifacts	Minimal artifacts	Prominent artifacts	Major artifacts	Severe artifacts
Image contrast	Very good to perfect contrast	Good to very good contrast	Medium contrast	Poor contrast	Inappropriate contrast

### Statistical data analysis

GraphPad Prism (version 6.0; GraphPad Software Inc.) and SPSS (version 25.0; IBM SPSS Statistics for Windows, IBM Corp.) were used for statistical data analyses. The level of statistical significance was set at *p* < 0.05.

For patient details, scanning parameters and dose characteristics, and scores assigned by the readers, descriptive statistics were calculated. Furthermore, the number of segments with reported degenerative changes and the absolute frequency of each specific degenerative change was counted. The number of any missed degenerative changes when compared with consensus reading of the SD scans was noted. Analyses were performed separately for the evaluations of R1 and R2 and for all reconstructed image data, respectively.

To compare overall image quality, overall artifacts, and image contrast of MDCT with virtually lowered tube current or sparse sampling against SD scanning, Wilcoxon matched-pairs signed-rank tests were performed (D100P100 vs. D50P100/D10P100/D5P100/D3P100 and D100P50/D100P10/D100P5/D100P3 for R1 and R2, respectively). Moreover, Wilcoxon matched-pairs signed-rank tests were also conducted between MDCT with virtually lowered tube current or sparse sampling at each level of reduction (D50P100 vs. D100P50, D10P100 vs. D100P10, D5P100 vs. D100P5, and D3P100 vs. D100P3 for R1 and R2, respectively). Intraclass correlation coefficients (ICCs) were computed to assess inter-reader agreement (two-way mixed model).

## Results

### Cohort characteristics

**Fig. 1:**
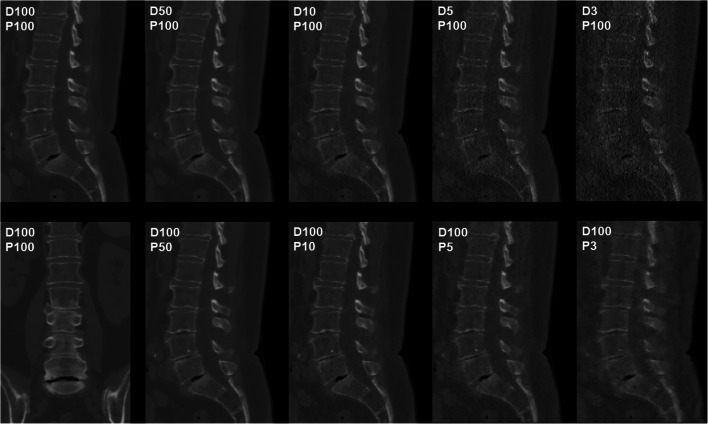
Patient case 1—lumbar degenerative changes (bone window). This figure depicts the lumbosacral spine of a 61-year-old female patient in standard-dose (SD) scans (D100P100, sagittal and coronal plane, bone window) and in simulated scans with virtual reduction of tube current (D50P100, D10P100, D5P100, and D3P100, sagittal plane, bone window) or sparse sampling (D100P50, D100P10, D100P5, and D100P3, sagittal plane, bone window). For image evaluation, all planes were available to the readers with individually adaptable windowing options. The patient showed multi-segmental non-calcified disc herniations and spondylosis according to original imaging

**Fig. 2:**
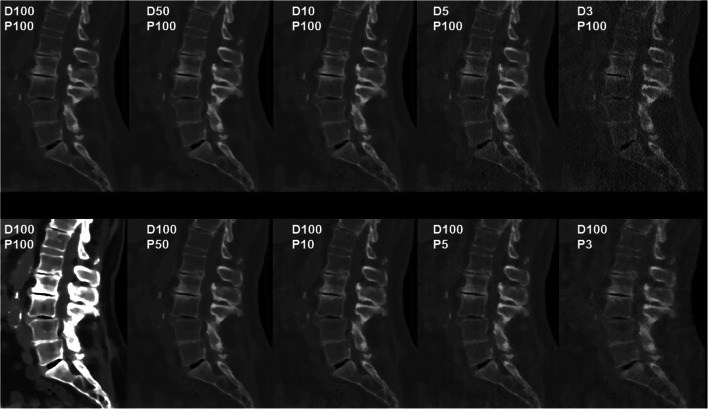
Patient case 2—lumbar degenerative changes (bone window). This figure shows the lumbosacral spine of an 82-year-old female patient in standard-dose (SD) scans (D100P100, sagittal plane, bone and soft tissue window) and in simulated scans with virtual reduction of tube current (D50P100, D10P100, D5P100, and D3P100, sagittal plane, bone window) or sparse sampling (D100P50, D100P10, D100P5, and D100P3, sagittal plane, bone window). For image evaluation, all planes were available to the readers with individually adaptable windowing options. The patient showed multi-segmental non-calcified disc herniations, spondylosis, and pseudospondylolisthesis (L3/4) according to original imaging

**Fig. 3:**
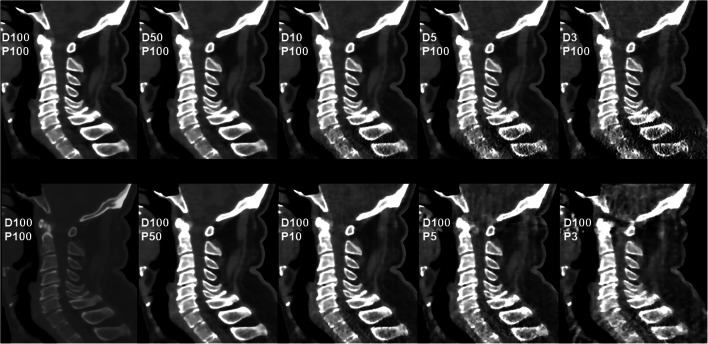
Patient case 3—cervical degenerative changes (soft tissue window). This figure depicts the cervical spine of a 63-year-old male patient in standard-dose (SD) scans (D100P100, sagittal plane, bone and soft tissue window) and in simulated scans with virtual reduction of tube current (D50P100, D10P100, D5P100, and D3P100, sagittal plane, soft tissue window) or sparse sampling (D100P50, D100P10, D100P5, and D100P3, sagittal plane, soft tissue window). For image evaluation, all planes were available to the readers with individually adaptable windowing options. The patient showed multi-segmental non-calcified disc herniations and spondylosis according to original imaging

**Fig. 4:**
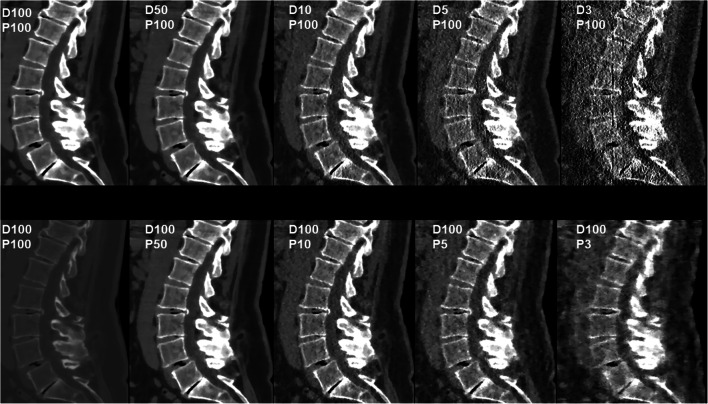
Patient case 4—lumbar degenerative changes (soft tissue window). This figure shows the lumbar spine of a 66-year-old female patient in standard-dose (SD) scans (D100P100, sagittal plane, bone and soft tissue window) and in simulated scans with virtual reduction of tube current (D50P100, D10P100, D5P100, and D3P100, sagittal plane, soft tissue window) or sparse sampling (D100P50, D100P10, D100P5, and D100P3, sagittal plane, soft tissue window). For image evaluation, all planes were available to the readers with individually adaptable windowing options. The patient showed multi-segmental disc herniations (with calcified components L2/3), spondylosis, and pseudospondylolisthesis (L4/5) according to original imaging

**Fig. 5:**
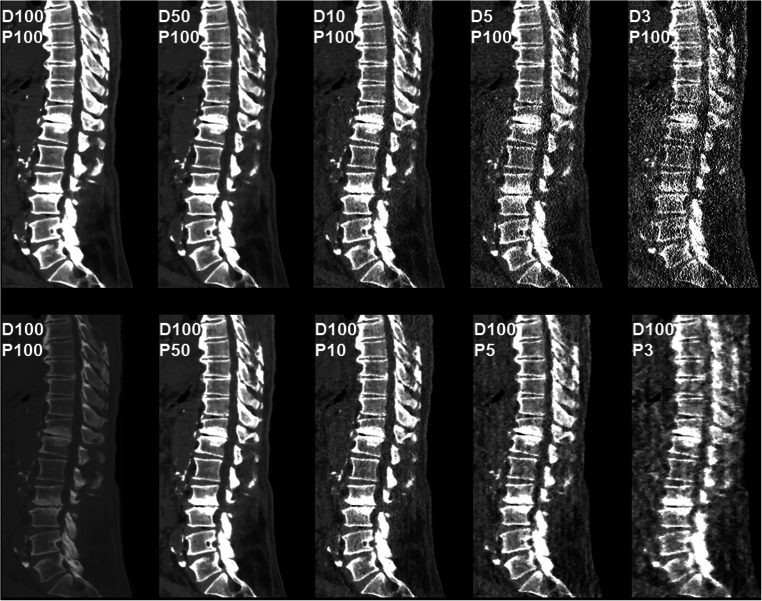
Patient case 5—lumbar degenerative changes (soft tissue window). This figure depicts the lower thoracic to lumbosacral spine of a 59-year-old male patient in standard-dose (SD) scans (D100P100, sagittal plane, bone and soft tissue window) and in simulated scans with virtual reduction of tube current (D50P100, D10P100, D5P100, and D3P100, sagittal plane, soft tissue window) or sparse sampling (D100P50, D100P10, D100P5, and D100P3, sagittal plane, soft tissue window). For image evaluation, all planes were available to the readers with individually adaptable windowing options. The patient showed multi-segmental non-calcified disc herniations and spondylosis according to original imaging

Image data of 26 patients (mean age: 68.9 ± 11.7 years, range: 37.5–86.9 years, 42.3% males) were used in this study, with simulations of sparse-sampled MDCT or MDCT with lowered tube current being available from all patients. Scans covered the cervical spine (in 23.1% of patients) or lumbosacral spine (in 76.9% of patients), with a median of 6.5 segments being included in the FOV (range: 3–10 segments) (Figs. 1, 2, 3, 4, and 5).

In total, degenerative spine disease affected 84 segments when considering the whole cohort. Spondylosis was most common (68 incidences, 50% of detected degenerative changes), followed by non-calcified disc herniation (52 incidences, 38.2% of detected degenerative changes) and pseudospondylolisthesis (8 incidences, 5.8% of detected degenerative changes, 100% Meyerding grade I) as well as spondylolisthesis (with spondylolysis; 4 incidences, 3.0% of detected degenerative changes, 50% Meyerding grade I and 50% Meyerding grade II). Calcified disc herniation (4 incidences, 3.0% of detected degenerative changes) occurred infrequently.

### Qualitative image analysis

Compared with SD scanning, both virtual tube current reduction and sparse sampling decreased overall image quality and image contrast and increased overall artifacts, with significant differences (*p* < 0.05) between SD MDCT and all levels of virtually lowered tube current or sparse sampling except for D100P50 (overall image quality: R2: *p* = 0.25; overall artifacts: R2: *p* = 0.50; image contrast: R1: *p* = 0.06, R2: *p* = 0.13).

However, sparse-sampled MDCT at 50%, 10%, and 5% of original projections showed better results when compared with MDCT with virtual reduction of tube current by 50%, 10%, and 5% (Table 3). Specifically, imaging data of D100P10 still showed good to very good image quality and contrast with only minimal artifacts (Table 3). Agreement between readers was fair to excellent for overall image quality (ICC range: 0.40–0.91), good to excellent for overall artifacts (ICC range: 0.65–0.92), and good to excellent for image contrast (ICC range: 0.84–0.99; Table 3).

**Table 3:** Results of qualitative image evaluation

	D100P100	D50P100	D100P50	*p*	D10P100	D100P10	*p*	D5P100	D100P5	*p*	D3P100	D100P3	*p*
Overall image quality													
R1	1.0	1.4 ± 0.5	1.3 ± 0.5	n.s.	2.7 ± 0.6	2.5 ± 0.5	n.s.	3.7 ± 0.8	3.5 ± 0.6	n.s.	4.5 ± 0.7	4.7 ± 0.6	n.s.
R2		1.3 ± 0.5	1.1 ± 0.3	n.s.	2.5 ± 0.6	2.2 ± 0.4	*0.039*	3.5 ± 0.5	3.2 ± 0.4	*0.022*	4.4 ± 0.6	4.4 ± 0.5	n.s.
ICC	–	0.83	0.47	–	0.86	0.40	–	0.82	0.66	–	0.91	0.66	–
Overall artifacts													
R1	1.0	1.4 ± 0.5	1.2 ± 0.4	n.s.	2.7 ± 0.6	2.5 ± 0.5	n.s.	3.7 ± 0.7	3.5 ± 0.6	n.s.	4.5 ± 0.7	4.7 ± 0.6	n.s.
R2		1.4 ± 0.5	1.1 ± 0.3	*0.016*	2.5 ± 0.5	2.2 ± 0.4	*0.016*	3.5 ± 0.5	3.4 ± 0.5	n.s.	4.5 ± 0.5	4.5 ± 0.5	n.s.
ICC	–	0.92	0.65	–	0.80	0.68	–	0.86	0.81	–	0.90	0.77	–
Image contrast													
R1	1.0	1.5 ± 0.5	1.2 ± 0.4	n.s.	2.7 ± 0.7	2.5 ± 0.5	n.s.	3.6 ± 0.8	3.5 ± 0.6	n.s.	4.4 ± 0.9	4.7 ± 0.6	n.s.
R2		1.6 ± 0.5	1.2 ± 0.4	*0.003*	2.7 ± 0.7	2.3 ± 0.5	*0.037*	3.7 ± 0.7	3.4 ± 0.6	n.s.	4.4 ± 0.9	4.6 ± 0.6	n.s.
ICC	–	0.88	0.93	–	0.99	0.84	–	0.93	0.97	–	0.99	0.97	–

This table shows the results of qualitative image evaluation by reader 1 (R1) and reader 2 (R2) for overall image quality, overall artifacts, and image contrast (based on 5-point Likert scales). The standard-dose (SD) scans (D100P100) were evaluated in consensus reading; scans with virtual reduction of tube current (D50P100, D10P100, D5P100, and D3P100) or sparse sampling (D100P50, D100P10, D100P5, and D100P3) were evaluated independently. Statistical iterative reconstruction (SIR) was used in all imaging data. Scores as mean ± standard deviation are depicted; *p* values indicate statistical significance (values < 0.05 are given in italics; *n.s.* not statistically significant) as based on comparisons between imaging with virtually lowered tube current or sparse sampling at each level of reduction (D50P100 vs. D100P50, D10P100 vs. D100P10, D5P100 vs. D100P5, and D3P100 vs. D100P3 for R1 and R2, respectively). Intraclass correlation coefficients (ICCs) are given for inter-reader agreement

A 50% reduction of tube current or projection numbers still allowed for correct detection of all degenerative changes (Table 4). Furthermore, using sparse-sampled MDCT with 10% of original projection numbers still allowed for detection of all segments affected by degenerative spine disease, in contrast to MDCT with virtual tube currents of 10% of original imaging (Table 4). All specific degenerative changes were detected by both readers for D100P10, whereas readers missed a considerable fraction of herniated discs in MDCT with virtual reduction of tube current by 90% (D10P100: non-calcified disc herniation: R1: 12 out of 52 missed, R2: 11 out of 52 missed; Table 4).

**Table 4:** Segments and characteristics of degenerative changes

		D100P100	D50P100	D100P50	D10P100	D100P10	D5P100	D100P5	D3P100	D100P3
Segments with detected degenerative changes										
Number of segments	R1	84	84	84	82 *(2)*	84	76 *(8)*	78 *(6)*	71 *(13)*	73 *(11)*
	R2		84	84	82 *(2)*	84	75 *(9)*	79 *(5)*	72 *(12)*	72 *(12)*
Specifics of degenerative changes										
Spondylosis	R1	68	68	68	68	68	68	68	68	68
	R2		68	68	68	68	68	68	66 *(2)*	68
Pseudospondylolisthesis	R1	8	8	8	8	8	8	8	5 *(3)*	5 *(3)*
	R2		8	8	8	8	8	8	5 *(3)*	5 *(3)*
Spondylolisthesis (with spondylolysis)	R1	4	4	4	4	4	4	4	4	4
	R2		4	4	4	4	4	4	4	4
Non-calcified disc herniation	R1	52	52	52	40 *(12)*	52	14 *(38)*	36 *(16)*	1 *(51)*	4 *(48)*
	R2		52	52	41 *(11)*	52	15 *(37)*	27 *(25)*	2 *(50)*	9 *(43)*
Calcified disc herniation	R1	4	4	4	4	4	4	4	4	4
	R2		4	4	4	4	4	4	4	4

This table shows the number of segments with detected degenerative changes and the number of specific changes for the whole cohort according to reader 1 (R1) and reader 2 (R2). The standard-dose (SD) scans (D100P100) were evaluated in consensus reading; scans with virtual reduction of tube current (D50P100, D10P100, D5P100, and D3P100) or sparse sampling (D100P50, D100P10, D100P5, and D100P3) were evaluated independently. Italic numbers in brackets represent the number of missed degenerative changes when compared with SD imaging

### Radiation dose

The mean volumetric CT dose index (CTDI_vol_) for SD scanning was 13.8 ± 5.0 mGy (range: 8.6–27.4 mGy), and it amounted to 6.9 mGy (D50P100 or D100P50), 1.4 mGy (D10P100 or D100P10), 0.7 mGy (D5P100 or D100P5), and 0.4 mGy (D3P100 or D100P3) for simulated MDCT with tube current reduction or sparse sampling on average, respectively.

## Discussion

This study applied stepwise simulations of lowered tube currents and sparse sampling combined with SIR for MDCT in patients with suspected or previously diagnosed degenerative spine disease. Both virtual lowering of tube currents as well as sparse sampling with SIR were feasible and enabled decreases in radiation doses. However, sparse sampling allowed for greater reductions in radiation exposure when compared to virtual tube current reduction, with MDCT with 10% of original projections still providing scans with sufficient image quality and diagnostic value since no degenerative changes were missed when compared with original imaging.

Previous literature on LD CT in patients with back pain with or without radiculopathy used lowering of tube currents for dose reductions [12–16]. These studies mostly compared patients who underwent different imaging protocols for CT of the cervical or lumbar spine [12, 14, 15]. Such studies were successful in reducing radiation exposure, but their study design by comparing mainly two groups of different patients for only two dose levels did not allow to systematically reveal a certain tube current threshold for diagnostic purpose, as this study can provide by using stepwise simulations on an intra-subject level. To the authors’ knowledge, only one previous study used a comparable approach (restrictions to 65%, 50%, 35%, and 20% of SD imaging), but only focused on disc herniations as one manifestation of spine degeneration and did not use advanced image reconstructions, most probably due to the non-availability at the time of publication [16]. For patients with suspected lumbar disc herniation, tube charge settings could be reduced to 65% of SD imaging in this previous study [16]. In contrast, results of the present study indicate that more dedicated tube current reductions by 50% might be possible without relevant restrictions for diagnostic purpose, evaluating a broader range of common degenerative changes at the spine. This may be due to the interim developments of MDCT technology including advancements in image reconstruction, such as SIR.

Sparse sampling is a novel approach that has potential to lower radiation exposure to the patient. It describes the reduction in projection images for scanning with the anticipated benefit of lowered overall energy [17, 18]. At the spine, previous research demonstrated that determination of bone mineral density (BMD) and microstructure of vertebrae using sparse-sampled MDCT is more robust compared with that using MDCT with lowered tube currents [27]. Additionally, images with up to 50% reductions in radiation dose through sparse sampling can be used for finite element (FE)-based predictions of femoral failure load [35]. Sufficient image quality and diagnostic accuracy for detection of vertebral fractures could be achieved with 50% of original projections, while, on the contrary, MDCT with 50% lowered tube currents yielded inferior results [28].

Concerning the degenerative spine, the present study suggests that both sparse sampling and virtual reductions of tube current have potential for dose reduction. In particular, sparse-sampled MDCT with only 10% of original projections (reduction of CTDI_vol_ from 13.8 to 1.4 mGy) generates scans entailing high diagnostic accuracy at sufficient image quality for diagnostic purposes. Thus, when compared with MDCT with virtually lowered tube current, sparse sampling could enable even greater reductions in radiation exposure while both readers were able to identify all degenerative changes without any missed pathology when compared with SD MDCT. This seems to be primarily due to better detectability of non-calcified disc herniations in sparse-sampled MDCT when compared with MDCT with virtual tube current reduction. In this regard, particularly soft tissue contrast seems to be affected in LD scans, with image noise showing considerable increases particularly for virtually reduced tube current, thus hampering unequivocal identification of non-calcified disc herniations. For degenerative changes directly affecting the vertebrae like spondylosis, pseudospondylolisthesis or spondylolisthesis (with spondylolysis), as well as calcified disc herniations, virtual tube current reduction was not clearly inferior to sparse sampling. However, pseudospondylolisthesis was missed in scans simulated with only 3% of initial tube current or projection numbers, most likely due to fading of bony contours of the vertebrae and related issues with detectability of vertebral shift in the anterior-posterior direction. Of note, all patients with pseudospondylolisthesis of this study only showed a minor degree of listhesis (Meyerding grade I), probably making clear detection of slight vertebral shift impossible for MDCT with very low doses.

The reason for the overall better results of sparse-sampled data most likely relates to the inherently different methods of sparse sampling and tube current reduction. Specifically, while the approach of tube current reduction leads to considerable increases in image noise that can negatively affect diagnostic use, sparse sampling implies maintained energy delivery for the individual projection image, but lowered overall radiation exposure due to the decrease in total projection numbers [17, 18]. Consequently, preserved image quality for the individual projection can be achieved while circumventing the influence of electronic readout noise, leading to largely preserved structural image information [17, 18].

Sparse sampling reduces the quantity of measurement during the MDCT exam, which can be compensated by advanced reconstruction algorithms. We used in-house-developed SIR for MDCT with virtually lowered tube currents and sparse sampling, respectively. Advanced reconstruction methods such as IR have potential to further suppress noise in imaging data, which increases image quality and can therefore allow for further restrictions in radiation exposure from scanning without relevant loss of structural image information [17, 19–23]. A study investigating the feasibility of SIR in predicting MDCT-based BMD and vertebral bone strength from FE analysis in comparison to data reconstructed with FBP revealed that SIR produced images of the best quality with regard to noise, signal-to-noise, and contrast-to-noise ratios [36]. Other previous research showed that IR at certain levels can improve image quality of LD CT of the lumbar spine when compared with FBP [37]. Similarly, LD CT of the cervical spine using IR was evaluated, recommending that rather high strength levels of IR might be used for visualization of intervertebral discs and the content of neural foramina [13]. In accordance with these findings, the present study also used a high level of regularization as this was considered superior for evaluations of the degenerative spine following consensus evaluation of different regularization levels. However, most previous work applied a hybrid IR algorithm [12, 13, 15]. Hybrid IR algorithms are generally characterized by reasonable reconstruction speed but are less effective in artifact and noise reduction, with more advanced approaches, such as model-based IR or SIR, facilitating further improvement in image quality [17, 19, 20].

This study also has limitations that should be considered. First, this study only investigated degenerative changes, thus not including pathologies with osseodestructive behavior like spondylodiscitis or bone metastases. Such pathologies can share similar symptoms with degenerative spine diseases and may represent important differential diagnoses. Thus, studies investigating LD MDCT by means of virtual tube current reduction and, importantly, sparse sampling are needed to expand clinical applicability of our approach. Second, LD MDCT was only simulated in this study, which was based on raw data taken from SD MDCT. This is related to the circumstance that MDCT systems capable of employing sparse sampling have not yet been made commercially available, hampering our approach to be seamlessly introduced into the clinical routine. Sparse sampling requires additional controlling units in the X-ray tube, increasing stability demands, and may increase the total cost of the system. Leading MDCT manufacturers have not yet shown sufficient interest in further systematic investigation and extra implementation of the technique, although first prototypes of systems have been developed successfully [38, 39]. Yet, distribution of sparse-sampled imaging beyond mere application in the research setting may become possible soon.

In conclusion, tube current reduction and sparse sampling combined with SIR enable decreases in radiation exposure for MDCT of the degenerative spine. However, sparse-sampled MDCT with SIR could lead to greater reductions in radiation exposure, with a 90% decrease in projection numbers (reduction of CTDI_vol_ from 13.8 to 1.4 mGy) when compared with original imaging still providing sufficient image quality and high diagnostic value since no degenerative changes were missed in this study. Thus, particularly sparse sampling combined with advanced image reconstruction might be a promising option for LD MDCT in patients with suspected degenerative spine disease.

## Abbreviations

BMD: Bone mineral density
CT: Computed tomography
CTDI_vol_: Volumetric CT dose index
FBP: Filtered back projection
FE: Finite element
FOV: Field of view
ICC: Intraclass correlation coefficient
IR: Iterative reconstruction
LD: Low dose
MDCT: Multi-detector CT
MRI: Magnetic resonance imaging
PACS: Picture archiving and communication system
R1: Reader 1
R2: Reader 2
SD: Standard dose
SIR: Statistical IR
